# Anti-cancer activities of pH- or heat-modified pectin

**DOI:** 10.3389/fphar.2013.00128

**Published:** 2013-10-08

**Authors:** Lionel Leclere, Pierre Van Cutsem, Carine Michiels

**Affiliations:** ^1^Unité de Recherche en Biologie Cellulaire, Namur Research Institute for Life Sciences, University of NamurNamur, Belgium; ^2^Unité de Recherche en Biologie Cellulaire Végétale, University of NamurNamur, Belgium

**Keywords:** pectin, cancer, galectin-3, drug combination, apoptosis, chemoprevention

## Abstract

Despite enormous efforts that have been made in the search for novel drugs and treatments, cancer continues to be a major public health problem. Moreover, the emergence of resistance to cancer chemotherapy often prevents complete remission. Researchers have thus turned to natural products mainly from plant origin to circumvent resistance. Pectin and pH- or heat-modified pectin have demonstrated chemopreventive and antitumoral activities against some aggressive and recurrent cancers. The focus of this review is to describe how pectin and modified pectin display these activities and what are the possible underlying mechanisms. The failure of conventional chemotherapy to reduce mortality as well as serious side effects make natural products, such as pectin-derived products, ideal candidates for exerting synergism in combination with conventional anticancer drugs.

Despite enormous progress in oncology therapy during the last decade, especially regarding the development of “smart drugs,” cancer still remains one of the leading causes of death. Hence, the development of new therapeutic strategies remains a high priority. Natural compounds represent an important source of new “leads” with potent chemotherapeutic or chemopreventive activity. Structure-activity relationship studies have led to the development of natural molecules or of semi-synthetic analogs with higher activity or lower toxicity. Two of the best examples currently used in cancer therapy are paclitaxel and etoposide. In this review, we will describe what is known about one particular class of complex plant polysaccharides, pectin, and its potential anti-cancer activities.

## DESCRIPTION OF PECTIN

In 1825, a French chemist and pharmacist, Henri Braconnot, who was an expert in the extraction of active components from plants, was the first to discover a heteropolysaccharide with gelling properties which he named “pectic acid” (in ancient Greek πηκτ ικóς meaning coagulant).

Pectin is a family of complex polysaccharides, which are found in high amounts in plant primary wall. The main role of the plant wall components is to give mechanical strength to plants, to maintain an extracellular water phase by imbibition and to provide a barrier from external environment.

The exact chemical structure of pectin is still under debate. Pectins are a family of covalently linked galacturonic acid-rich polymers. Until now, three main pectic polysaccharides have been isolated from plant wall whose structure has been identified. They are homogalacturonan (HG), rhamnogalacturonan-I (RG-I) and substituted galacturonans (GS).

**FIGURE 1:**
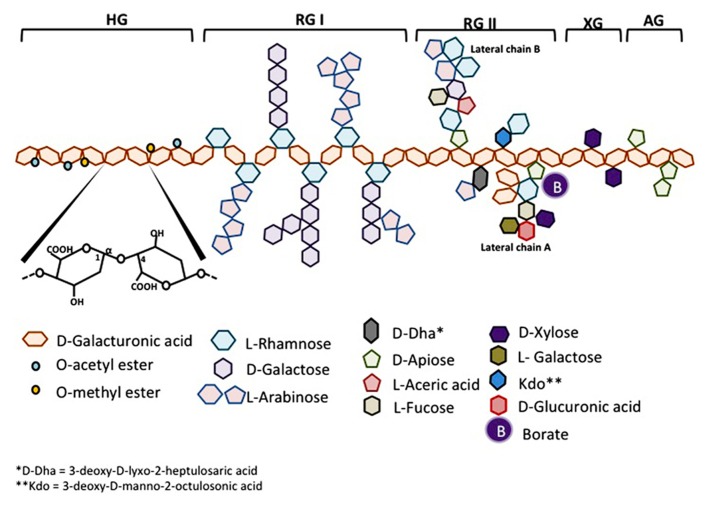
**Schematic representation of pectin structure**. AG, arabinogalactan; HG, homogalacturonan; RG, rhamnogalacturonan; XG, xylogalacturonan.

Homogalacturonan which constitutes about 65% of pectin molecule is a linear chain of D-galactopyranosyluronic acid (Gal*p*A) bound in α-1,4. The carboxyl group of some residues can be methyl-esterified. According to the plant species, HGs may also be partly O-acetylated on C-3 or C-2 (**Figure 1**).

Rhamnogalacturonan-I makes about 20–35% of pectin. RG-I is a family of pectic polysaccharides whose main chain is a repetition of disaccharides composed of galacturonic acid and rhamnosyl bound [→4)-α-D-Gal*p*A-(1→2)-α-L-Rhap-(1→]. The Gal*p* residues forming the main chain can be O-acetylated in C-3 or C-2 but are usually not linked with monomers or lateral chains. According to the plant species, about 20–80% of the rhamnosyl residues are substituted with neutral or acidic oligosaccharide chains on the carbon C4 of rhamnosyl residues. The most frequent lateral chains contain α-L-arabinofuranosyl (Ara*f*) and/or galactopyranosyl (Gal*p*). These lateral chains (arabinans, galactans or arabinogalactans) may be linear or branched (**Figure 1**).

Substituted galacturonans make a group of various polysaccharides whose linear chain is composed of D-Gal*p*A residues linked in α-1,4 (as in HG) and on which are grafted other residues. Among these GS, is rhamnogalacturonan-II (RG-II). RG-II has nothing to do with RG-I, its main chain is not composed of GalA-Rhap disaccharide but of a HG chain. Four types of chains with structurally different oligosaccharides are linked to the main chain of RG-II, they are composed of 12 types of glycosyl residues bound together by at least 22 types of glycosidic bounds. One nonasaccharide (lateral chain B) and one octasaccharide (lateral chain A) are attached in C-2 of some GalA residues from the main chain and two different disaccharides are linked in C-3 of the main chain. The localization of these lateral chains one in relation to the other is not yet determined (**Figure 1**). RG-II is often found in dimers thanks to a borate ion located on chain A. This dimerisation seems essential for the integrity of the plant cell wall. Despite its complexity, the RG-11 structure is well conserved in vascularized plants. Very few mutants with modified RG-II have been identified until now, which indicates the importance to conserve its structure. Other GS have been described in a short number of plants. Xylogalacturonan contains β-D-xylosyl (Xylp) linked in C3 of the main chain and is present in reproductory tissues of plants like apple, carrot and cotton. Apiogalacturonan contains monomers or dimers of β-D-apioduranosyl (Apif) attached in C-2 and C-3 of the main chain. Apiogalacturonan is found in some monocotyledons (Ridley et al., 2001; Mohnen, 2008; Caffall and Mohnen, 2009; Harholt et al., 2010; **Figure 1**).

The most accepted model for pectin structure is a main backbone of HG in which are intercalated regions of RG-I, RG-II and GS (Caffall and Mohnen, 2009). There are linkages between pectin polysaccharides as well as to other wall molecules, combined to make a network that makes the primary cell wall.

### FUNCTION OF PECTIN IN PLANT CELL WALL

As mentioned here above, the role of the components of the plant cell wall is first to give mechanical solidity and to form a barrier from the external environment. HGs and RGII are known to be responsible for the wall rigidification. HGs have the property to form structures which are named “egg boxes.” Two HG chains are bound one to the other through interactions including bivalent Ca^2^^+^ ions intercalated in between them (Liners et al., 1989). This process is important for the gelling of pectin.

The mechanical role of RG-I has been less studied but it seems that RG-I may play a role in cell wall plasticity, for example by preventing HG chains to interact with Ca^2^^+^ ions. Transgenic plants with decreased amounts of arabinans and galactans display a stiffening of their cell wall.

Pectin organization and composition in plant primary cell wall depend on the growth state of the plant, on the tissues and on the plant species. Its synthesis is a complex process involving numerous enzymes that are just becoming to be identified (Atmodjo et al., 2013).

### BIOLOGICAL ACTIVITIES OF OLIGOGALACTURONIDES IN PLANTS

There are three different ways for a pathogen to enter into a plant: to go through a natural opening, such as stomata; to seep into a wound or to digest the cell wall. Pectin is then the first substrate. Pathogens are able to secrete endopolygalacturonases and endopectate lyases which degrade HGs present in the cell wall and then release oligogalacturonides (OGAs). OGAs are biologically active carbonhydrates which act as signal molecules initiating defense responses from the plant. The first defense response observed in response to OGA production is the production of reactive oxygen species such as H_2_O_2_ and $O_{2}^{-}$. OGAs also initiate signaling pathways that activate defense systems in plants, like the production of protease inhibitors able to block the activity of proteases secreted by insects to digest plant cell wall. Finally OGAs are also responsible for wall reinforcement in response to pathogen infection. In addition to their roles in plant defense systems, OGAs also influence plant growth and development and they play a role in fruit ripening.

### PECTIN ACTIVITIES IN HUMAN BEINGS

Since humans are able to extract pectin, they try to use its huge potential to their benefits. In addition to be used as a gelling agent in food industry, pectin displays properties useful in medicine (Lattimer and Haub, 2010). In humans, pectin, as a dietary fiber, is not enzymatically digested in the small intestine but is degraded by microbia in colon. It keeps its gelling action in the digestive track, so that it slows down digestion. This is very beneficial in patients with Dumping syndrome who have a too rapid digestion within their stomach (Lawaetz et al., 1983). Pectin is also capable of diminishing blood cholesterol level and of stimulating lipid excretion. However, the exact mechanisms underlying these effects are not known yet (Brown et al., 1999). Pectin is also investigated for its ability to increase ^137^Cs clearance (Nesterenko et al., 2004). ^137^Cs is a radio-isotope produced during uranium fission that is found in Tchernobyl area. *PectaSol*®, a modified form of pectin, when eaten during several days, allows a better clearance via the urinary track of toxic elements like arsenic or cadmium, which seem to be chelated by modified pectin and then eliminated in the urine (Eliaz et al., 2006). Finally, several studies have shown that orally taken pectin decreases the risk of intestinal infection and of diarrhea in children by favoring the growth of “good” bacteria in the colon (e.g., *Biffidobacteria* and *Lactobacillus*) to the detriment of pathogenic bacteria (Olano-Martin et al., 2002).

## PECTIN AND CANCER, STATE OF THE ART

Pectin is known for its anti-tumor activities already since several decades. Because of its highly complex structure, it is not surprising that it displays so many different biological activities (Maxwell et al., 2012). In the literature, it is not easy to make the link between structure and pectin bioactivity, notably because the origin of the pectin used in the different studies and the possible chemical modifications that create molecular fragments it has undergone are not always well described. It has to be noted that differences in size of the fragments generated, in their degree of esterification (DE), in the nature of the sugar monomers present in the polysaccharide(s) and the extraction procedure are likely to have significant influence on the properties on these different types of pectin. However, six main issues will be highlighted hereunder.

### EFFECT OF PECTIN AS A DIETARY FIBER

As a dietary fiber, pectin plays a role in preventing colon cancer. In 1979, Watanabe et al. (1979) have shown that rats treated with azoxymethane or methylnitrosourea develop less colon tumors if their diet was enriched in pectin. Heitman et al. (1992) similarly demonstrated less numerous colon tumors in rats treated with 1,2-dimethylhydrazine if they were given pectin. Ohkami et al. (1995) evidenced that citrus and apple pectin in the diet of rats exposed to azoxymethane decreased carcinogenesis. The two types of pectin decreased the number of tumors and apple pectin decreased the activity of β-glucuronidase, an enzyme from fecal bacteria whose activity is correlated to colon cancer development (Ohkami et al., 1995). Different types of carbohydrates have been studied for their antimutagenic activity. For example, Hensel and Meier (1999) showed that xyloglucans and rhamnogalacturonans decreased the mutagenic effect of 1-nitropyrene. This protection is dose-dependent and could come from a direct interaction between cells and polymers that would protect the cells from mutagenic effects of 1-nitropyrene.

Colon carcinogenesis is a multi-step process that results from disruption of the balance between proliferation of colonocytes at the base of the crypt and loss of colonocytes at the luminal surface due to apoptosis. Most colon cancer cells become resistant to apoptosis, hence promoting tumor growth. Chemoprotection may arise if luminal colonocyte sensitivity to apoptosis is restored. Schwartz’s team first showed that in rats, a pectin rich diet, compared to a standard diet, favored the expression of caspase-1 in luminal colonocytes from colon crypts and increased cleaved PARP level in basal and luminal colonocytes. The expression of the anti-apoptotic protein Bcl2 is on the other hand higher in rats with standard diet (Avivi-Green et al., 2000c). They then demonstrated that the activation of apoptosis due to the pectin rich diet had protective effects and diminished the number and the size of tumors in rats treated with 1,2-dimethylhydrazine. Colonocytes of rats nourished with pectin presented a high activity of caspase-1 and expressed pro-caspase-3 at a higher level, with a higher level of cleaved PARP. Pectin *per se* may induce apoptosis since the viability of cells exposed in culture to different pectin-derived oligosaccharides is decreased. Olano-Martin et al. (2003) evidenced that when colon adenocarcinoma HT29 cells were incubated in the presence of pectin oligosaccharides during 3 days, an increase in apoptosis, in DNA fragmentation and in caspase-3 activity was observed. This is also true for cells from other types of cancer: Attari et al. (2009) demonstrated that concentrations of 100 μg/ml to 1 mg/ml of pectic acids induced apoptosis in rat GH3/B6 pituitary tumor cells in a concentration dependent way while concentrations of 2.5 and 5.0 mg/ml induced necrosis. DNA fragmentation which was directly proportional to the number of apoptotic cells was observed (Attari et al., 2009). In combination with n-3 polyunsaturated fatty acid-rich fish oil, pectin also demonstrated chemoprevention in a colon cancer model of rats injected with azoxymethane. This was associated with a decrease in Bcl-2 expression due to promoter methylation (Cho et al., 2012) as well as to changes in the expression profile of mRNA implicated in and of miRNA targeting canonical oncogenic signaling pathway (Davidson et al., 2009; Cho et al., 2011; Shah et al., 2011).

On the other hand, colonocyte apoptosis activation in animals fed with pectin is also largely due to butyrate, a molecule coming from pectin fermentation by colon bacteria flora (Avivi-Green et al., 2000a,b). Indeed, intracolonic instillation of butyrate recapitulates the effect of orally administered pectin (Avivi-Green et al., 2000b). Butyrate is also able to induce apoptosis in colonocytes *in vitro* in a p53-independent manner (Kolar et al., 2007) and by inducing mitochondrial Ca^2+^ overload (Kolar et al., 2011). In parallel, both *in vitro* in rat intestinal epithelial cells exposed to butyrate and in mice fed with a diet supplemented with 20% pectin, TGF-ß signaling has been demonstrated to be enhanced, leading to colonocyte growth inhibition and apoptosis. Apoptosis seems to be induced via an increased expression of Id2 (inhibitor of differentiation 2), probably via inhibition of selective isoforms of HDACs (Cao et al., 2011).

### ANTI-TUMOR ACTIVITY OF pH-MODIFIED PECTIN

Pectin can be modified by treatment at different pHs; the most studied pH-modified pectin is the one isolated from citrus (MCP, modified citrus pectin). pH-modification involves an alkaline treatment that causes ß-elimination reactions, which results in depolymerization of the polysaccharide backbone and de-esterification of the HG regions. This is followed by an acid treatment that cleaves neutral sugars, releases the branched regions of the pectin backbone and preferentially removes arabinose residues. Thus, arabinogalactans and galactans are generated in high amounts.

Modified citrus pectin was mainly studied in Avraham Raz’s laboratory and has shown strong anti-cancer activities. Injection of pectin increased the number of tumors detected in lung after B16-F1 melanoma cells implantation in C57BL/6 mice probably by increasing homotypic aggregation between tumor cells while MCP significantly diminished the number of metastases. MCP which is rich in galactoside residues seems to impair cell-cell interactions by competing with endogenous ligands of “galactoside binding proteins” and more particularly of galectin-3 (Platt and Raz, 1992; Inohara and Raz, 1994). Raz’s team also showed that orally administered MCP decreased the number of metastases in lung in rats injected with prostate cancer MAT-LyLu cells. This decrease was dose-dependent (Pienta et al., 1995). In 2002, they also evidenced that MCP decreased the growth of breast (MDA-MB-435) and colon (LSLiM6) tumors implanted in NRC nu/nu mice as well as the number of metastases in lung and lymph nodes. These effects were associated with anti-angiogenic effects since a decrease in the number of capillaries *in vivo* and an inhibition of tubulogenesis *in vitro* using HUVEC were observed (Nangia-Makker et al., 2002). Other works have also evidenced the anti-tumor activity of MCP. When added to the culture medium of prostate androgen-independent JCA-1 tumor cells, MCP diminished proliferation and tritiated thymidine incorporation. MCP decreased the expression of nm23, a protein whose expression is inversely correlated with metastasis in various cancers (Hsieh and Wu, 1995). Hayashi et al. (2000) showed that oral daily doses of 0.8 and 1.6 mg/ml MCP to Balb-C mice implanted with colon tumors decreased tumor size, of respectively 38 and 70%. GCS-100, which is a commercially available form of modified pectin, has been shown to be efficient against different lines of multiple myelomas some of them resistant to chemotherapy, by inducing caspase-3 and -8 activation as well as PARP cleavage. Modified pectin-induced apoptosis was partly inhibited by Z-VAD-fmk, a pan-caspase inhibitor (Chauhan et al., 2005). A phase II clinical study on prostate cancer patients showed that *PectaSol*® MCP significantly increased PSADT (PSA doubling time) in 7 out of the 10 cases included in this study (Guess et al., 2003). *PectaSol*® and its ameliorated version *PectaSol-C*® are cytotoxic for different cancer cell lines: LNCaP, PC3, CASP2.1, CASP1.1, and BPH-1. In CASP1.1 and PC3 cells, cytotoxicity was correlated with MAP kinase activation inhibition, increased Bim protein expression and caspase-3 cleavage (Yan and Katz, 2010). This product also inhibits the invasive behavior of human breast and prostate cancer cells *in vitro* (Jiang et al., 2013).

Galectin-3 seems to be a target of MCP. Galectin-3 protein can be found intra- and extracellularly and contains a lectin domain. It has pleiotropic functions, amongst which, it mediates cell-cell as well as cell-extracellular matrix adhesion, through binding to glycoconjugates. Indeed, this lectin-domain has a high affinity for ß-galactoside residues. Galectin-3 expression is dysregulated in transformed cells, being highly expressed in numerous different types of cancer cells (Newlaczyl and Yu, 2011). MCP has been shown to decrease liver metastasis in a mouse colon cancer model, in a dose-dependent manner. This effect may be linked to the higher expression of galactin-3 in the liver metastases (Liu et al., 2008). The relationship between MCP structure and its inhibitory activity on galectin-3 was investigated in several studies. One such example is the work by Sathisha et al. (2007) who compared the activation of pectins from different dietary plants. Pectins rich in galactose and arabinose and in arabinogalactan significantly inhibited galectin-3-dependent hemagglutination of MDA-MB-231 cells to erythrocytes (Sathisha et al., 2007). Pectin nearly mainly composed of RG-I isolated from okra, a tropical plant, arrested cell cycle of B16F10 cells in G2/M phase and induced apoptosis probably through interaction with galectin-3 (Vayssade et al., 2010). Gao et al. (2012) suggested that MCP ability to inhibit galectin-3 resides in its RG-I regions and more particularly from galactan, of which the nature of last residue is the most important. Gunning et al. (2013) confirmed that neutral galactan side chains did selectively bind to recombinant galectin-3. These active fragments can be obtained by enzymatic treatment of isolated RG-I regions from potato pectin (Gunning et al., 2009).

In conclusion, MCP displays many anti-metastatic properties demonstrated both *in vitro* and *in vivo*, in various malignancies. Many of them, if not all, are due to its binding to the pleiotropic galectin-3 protein which is overexpressed in cancer. Due to its well tolerance and among other plant-derived products, pectin-derived GCS-100 is being explored for the maintenance therapy of patient with B-chronic lymphocytic leukemia relapse (O’Brien and Kay, 2011).

### ANTI-TUMOR ACTIVITY OF OTHER FORMS OF MODIFIED PECTIN

Jackson et al. (2007) investigated the apoptosis induction of different forms of modified pectin in prostate cancer cells which were either androgen-dependent (LNCaP) or androgen-independent and which were not expressing galactin-3 (LNCaP C4-2). In their work, citrus pectin and pH-modified pectin, *PectaSol*®, exerted no pro-apoptotic activity while two different forms of heat-modified pectins, one commercially available and the other one prepared in their laboratory, did markedly induce apoptosis in the two cell lines (Jackson et al., 2007). They showed HGs, RG-I, and RG-II taken separately had no cytotoxic activity. Treatment of heat-modified pectin with pectinmethylesterase to remove galacturonosyl carboxymethylesters and/or with endopolygalacturonase to cleave non-methylesterified HG did not result in loss of activity. On the other hand, mild base treatment that removed ester bounds destroyed the pro-apoptotic activity. Biological effectiveness thus requires a base-sensitive linkage in OGAs other than a carboxymethylester bound. Size analyses of the active fragments suggested low mass (10–20 kDa) oligosaccharides (Jackson et al., 2007).

Similar results were obtained by Cheng et al. (2011) who tested the anti-tumor activity of different polysaccharide fractions isolated from ginseng on colon cancer HT-29 cells. While fractions rich in HG stopped cell cycle in G2/M phase, fractions rich in HG and modified by heat treatment exerted a much higher anti-proliferative activity, which was accompanied by caspase-3 activation and apoptosis induction (Cheng et al., 2011). Similarly, potato pectin, rich in HG, inhibited *in vitro* HT-29 cell proliferation and provoked a cell cycle arrest in G2/M phase. This inhibition was due to a decrease in cyclin B1 expression and in CDK-1 activity (Cheng et al., 2013). It is important to note that Kang et al. (2006) also produced a citrus pectin-derived oligosaccharide, which was biologically active, by irradiation, i.e., without chemical treatment. Pectin irradiated with 20 kGy and then dialyzed (WT <10,000) inhibited cancer cell growth.

### IMMUNOPOTENTIATING ACTIVITY OF PECTIN

Some of the components of pectin exert their anti-tumor activity *in vivo* by stimulating the immune system. Pectic polysaccharides named angelans and isolated from *Angelica gigas Nakai*, a Chinese medicinal plant, are immunopotentiators which increase immune functions of B lymphocytes, of macrophages and of “*natural* *killer”* cells and which directly activate T *helper* and cytotoxic lymphocytes. Angelans have also an anti-metastatic activity, it inhibits B16F10 cancer cell adhesion to the extracellular matrix as well as its invasion. In a urine model of colon cancer, apple oligogalactan (AOG) composed of five subunits showed preventive effects against the toxic and carcinogenic effects of 1,2-dimethylhydrazine and of sodium dextran sulfate. The underlying mechanisms included AOG targeting of the LPS/TLR4/NF-κB pathway by modifying TLR4 membrane distribution hence preventing LPS binding (Liu et al., 2010). HG-rich pectin polysaccharides isolated from red ginseng (*Ginseng panax*) exert both anti-tumor and immunomodulatory properties which derived from NO production by macrophages (Choi et al., 2008). In addition, this immunomodulatory effect ameliorates the paclitaxel-induced anti-cancer activity in mice with transplanted B16 melanoma tumors (Shin et al., 2004). As demonstrated by Chen et al. (2006) pectin effect on LPS-activated macrophages depends on its DE. Pectin esterified up to 90% (DE90) inhibits iNOS and COX2 expression in macrophages much more efficiently than pectin esterified to 30 or 60%. DE90 pectin also inhibits MAPK phosphorylation, IKK kinase activity as well as NF-κB and AP-1 activation. DE90 pectin binds LPS, which could modify LPS binding to its receptor (Chen et al., 2006). It has also been shown that *PectaSol-C* exerts immunostimulating activity in human blood, activating cytotoxic T cells, B cells and NK cells, inducing cytotoxicity toward chronic myeloid leukemia K562 cells (Ramachandran et al., 2011).

### MODIFIED PECTIN TO OVERCOME CHEMORESISTANCE

Chemoresistance is a heavy burden in the treatment of cancer, especially since a large number of patients already display metastatic disease at the time of diagnosis. The vast majority of anti-cancer drugs currently used act by inducing apoptosis via the intrinsic pathway. Numerous mechanisms underlie cancer chemoresistance (Rebucci and Michiels, 2013), but it appears that galectin-3 which is overexpressed in numerous tumor types, suppresses cell apoptosis and hence, decreases sensitivity of cancer cells to chemotherapeutic drugs (Glinsky and Raz, 2009). Since MCP has been shown to target galectin-3, several works were dedicated to delineate MCP-induced possible re-sensitization of cancer cells to different cytotoxic molecules. Johnson et al. (2007) showed that galectin-3 targeting via MCP or via a more specific inhibitor, lactosyl-L-leucine (LL), decreased malignant endothelial cell proliferation by themselves and sensitized these cells to the cytotoxic effect of doxorubicin. These two compounds also increases metastatic-derived MDA-MB-435 cells sensitivity to taxol both *in vitro* and *in vivo* (Glinsky et al., 2009). GCS-100, a commercially form of pH-MCP, enhanced bortezomide and dexamethasone-induced apoptosis in multiple myeloma cells and decreased viability. The effect was accompanied by a marked decrease in galectin-3 protein level (Chauhan et al., 2005). GCS-100 also induced calpain activation in prostate cancer cells that led to their sensitization to cisplatin treatment (Wang et al., 2010). Combination of modified pectin with different anti-cancer agents may thus represent an efficient new strategy to overcome resistance in cancer patients.

### USE OF PECTIN AS A VEHICLE FOR DRUG DELIVERY IN CANCER

Colon cancer is one of the most common cancers worldwide. Conventional chemotherapy is usually administered by intravenous injection to target tumor growth and metastases. However, severe side effects are observed. Oral administration using colon-specific delivery systems is expected to augment drug availability at tumor site while reducing systemic adverse effects. To this purpose, pectin-based vehicle is ideal since pectin is not digested in the gastrointestinal tract until it reaches colon, where it is fermented by residential bacteria, thus releasing the transported drug (for reviews, Chourasia and Jain, 2003; Patel et al., 2007; Wong et al., 2011). Different kinds of vehicles with different drugs and different chemistry binding or encapsulation have been designed and tested. Pectinate pellets or microspheres containing drugs led to prolonged dissolution and drug release in simulating colonic fluid (Zhang et al., 2011; Elyagoby et al., 2013). Other forms of vehicles like nanoparticles or pectin-based coated-based matrix tablets have been tested *in vitro* using colon cancer cells with efficient cell killing (Kanthamneni et al., 2010; Dev et al., 2011). Nanoparticles, capsules or microsponges have also been developed. Pharmacokinetics evaluation in rats and in rabbits provided evidence for colon delivery and delayed plasma appearance (Xu et al., 2005; Dev et al., 2011; Srivastava et al., 2012). However, actual demonstration for efficient treatment of colon cancer in animal models with pectin-based anti-cancer agent delivery vehicles is still lacking.

In parallel, pectin-derived biocompatible hydrogel loaded with different chemotherapeutic drugs have been produced. Doxorubicin-containing hydrogels displayed cytotoxicity toward HepG2 cells and inhibited homotypic aggregation of B16 melanoma cells, suggesting that it could also prevent metastasis *in vivo* (Takei et al., 2010). Similarly, pectin-coated chitosan gels encapsulating 5-fluorouracil showed controlled drug release and cytotoxicity against two cancer cell lines (Puga et al., 2013). Anti-cancer *in vivo* activity was also evidenced using doxorubicin-pectin hydrogel in subcutaneous B16 melanoma cell tumor in mice (Takei et al., 2013).

Finally, it is worth mentioning two other types of scaffolds including pectin- and fibrin-based nano-composites containing gemcitabine (Chandran et al., 2013) for the treatment of ovarian cancer and pectin nanoparticles loaded with methotrexate that displayed enhanced cytotoxicity toward HepG2 hepatocarcinoma cells *in vitro* (Chittasupho et al., 2013).

## CONCLUSION

**FIGURE 2:**
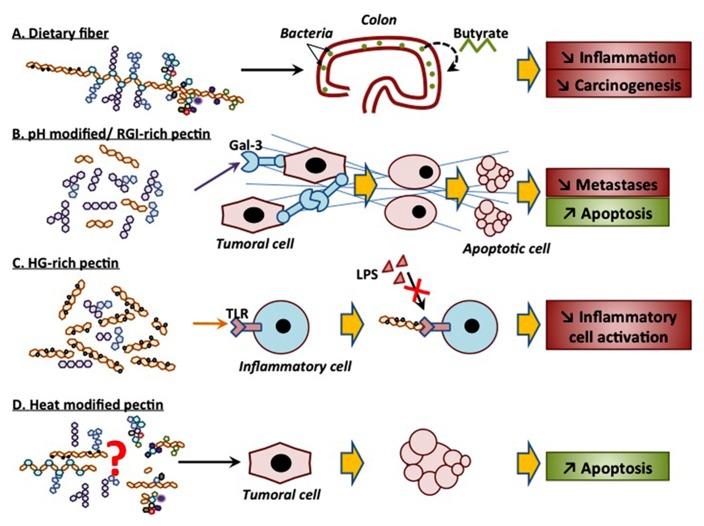
**Schematic representation of the different anti-cancer activities of different forms of pectin**.

In conclusion, pectin seems to exert anti-tumor activity on different cell lines and in different mice models, and this probably through different effects. These mechanisms depend on the structure of pectin or on the modified form of pectin that is likely to yield to various active fragments. Differences in extraction methods, in the plant species from which the pectin is isolated, in fragmentation techniques as well as in the structural complexity of pectin itself make the characterization of the active molecule(s) very difficult. The different anti-cancer activities of different forms of pectin are summarized in **Figure 2**. As a dietary fiber, pectin is not digested in the upper digestive tract and could protect cells from mutagenic attacks. In colon, pectin is fermented, by bacteria, into butyrate that inhibits colon inflammation and prevents carcinogenesis. pH-modified pectin as well as galactan-rich pectin (RG-I) are capable of interacting with galectin-3, thus inhibiting cell-cell interactions and cancer cell metastasis. Furthermore, HG-rich pectin with a high DE competes with LPS for TLR4 binding, hence preventing inflammatory cell activation. Finally, heat-modified pectin initiates apoptosis in cancer cells, in a galectin-3-independent manner. Despite the fact that the exact structure of these modified molecules is not yet known and that neither is their mechanisms of action, modified pectin emerges as one of the promising anti-metastatic drugs, especially if used in combination with more conventional molecules.

## Conflict of Interest Statement

The authors declare that the research was conducted in the absence of any commercial or financial relationships that could be construed as a potential conflict of interest.

## AUTHOR CONTRIBUTIONS

Lionel Leclere drafted the manuscript. Pierre Van Cutsem and Carine Michiels helped to write the final version of the manuscript. All authors read and approved the final manuscript.
